# Association of sleep problems with neuroendocrine hormones and coagulation factors in patients with acute myocardial infarction

**DOI:** 10.1186/s12872-018-0947-5

**Published:** 2018-11-21

**Authors:** Roland von Känel, Mary Princip, Jean-Paul Schmid, Jürgen Barth, Hansjörg Znoj, Ulrich Schnyder, Rebecca E. Meister-Langraf

**Affiliations:** 1 Department of Consultation-Liaison Psychiatry and Psychosomatic Medicine, University Hospital Zurich, Culmannstrasse 8, CH-8091 Zurich, Switzerland; 2 Department of Cardiology, Clinic Barmelweid, Barmelweid, Switzerland; 3 Complementary and Integrative Medicine, University of Zurich, Zurich, Switzerland; 4 Department of Clinical Psychology and Psychotherapy, University of Bern, Bern, Switzerland; 5 Medical Faculty, University of Zurich, Zurich, Switzerland; 6 Department of Psychiatry, Clienia Schlössli AG, Oetwil am See, Switzerland

**Keywords:** Acute coronary syndrome, Biomarker, Blood coagulation, HPA axis, Insomnia, Sleep apnea, Sympathetic nervous system

## Abstract

**Background:**

Obstructive sleep apnea (OSA) and insomnia are frequent sleep problems that are associated with poor prognosis in patients with coronary heart disease. The mechanisms linking poor sleep with an increased cardiovascular risk are incompletely understood. We examined whether a high risk of OSA as well as insomnia symptoms are associated with neuroendocrine hormones and coagulation factors in patients admitted with acute myocardial infarction.

**Methods:**

We assessed 190 patients (mean age 60 years, 83% men) in terms of OSA risk (STOP screening tool for the assessment of high vs. low OSA risk) and severity of insomnia symptoms (Jenkins Sleep Scale for the assessment of subjective sleep difficulties) within 48 h of an acute coronary intervention. Circulating concentrations of epinephrine, norepinephrine, cortisol, fibrinogen, D-dimer, and von Willebrand factor were measured the next morning. The association of OSA risk and insomnia symptoms with neuroendocrine hormones and coagulation factors was computed using multivariate models adjusting for demographic factors, health behaviors, somatic and psychiatric comorbidities, cardiac disease-related variables, and OSA risk in the model for insomnia symptoms, respectively, for insomnia symptoms in the model for OSA risk.

**Results:**

High OSA risk was identified in 41% of patients and clinically relevant insomnia symptoms were reported by 27% of patients. Compared to those with low OSA risk, patients with high OSA risk had lower levels of epinephrine (*p* = 0.015), norepinephrine (*p* = 0.049) and cortisol (*p* = 0.001). More severe insomnia symptoms were associated with higher levels of fibrinogen (*p* = 0.037), driven by difficulties initiating sleep, and with lower levels of norepinephrine (*p* = 0.024), driven by difficulties maintaining sleep.

**Conclusions:**

In patients with acute myocardial infarction, sleep problems are associated with neuroendocrine hormones and coagulation activity. The pattern of these relationships is not uniform for patients with a high risk of OSA and those with insomnia symptoms, and whether they contribute to adverse cardiovascular outcomes needs to be established.

**Trial registration:**

ClinicalTrials.gov NCT01781247.

## Background

Obstructive sleep apnea (OSA) is a common sleep-related breathing disorder that is caused by repeated upper airway obstruction during sleep and characterized through symptoms of snoring, breathing cessations while sleeping, and daytime sleepiness and fatigue [1]. Insomnia disorders are characterized by subjective sleep initiating and maintaining problems in spite of adequate circumstances to sleep and daytime impairment, whereas insomnia symptoms alone do not merit a formal diagnosis of insomnia [2].

Obstructive sleep apnea and insomnia symptoms, including difficulty initiating sleep, difficulty maintaining sleep and non-restorative sleep, have been associated with an increased risk of incident coronary heart disease (CHD) events, independently of a range of other risk factors [3–7]. Sleep problems may also be associated with poor prognosis of CHD. In two meta-analyses, CHD patients with OSA had an almost two-fold higher risk of poor cardiovascular prognosis, including recurrent CHD events and stroke, than CHD patients without OSA [8, 9]. In terms of insomnia symptoms in patients with acute myocardial infarction (MI), not feeling well-rested was predictive of case fatality in the 4 weeks post-MI in men and of 10-year risk of new CVD events in women [10]. In women, assessed 3–6 months after acute coronary syndrome (ACS), a sleep quality index, defined by insomnia symptoms was a predictor of recurrent CHD events at 5-year follow-up, after controlling for CVD risk factors and depressive symptoms [11]. In men who had undergone percutaneous coronary angioplasty, a state of vital exhaustion, characterized by subjective sleep disturbances, including troubles falling asleep, waking up repeatedly during the night, and not feeling well rested, along with profound feelings of fatigue, was predictive of new CHD events after a follow-up of 1.5 years [12].

Different biological mechanisms, initiated by intermittent hypoxia, are thought to underpin the association of OSA with an increased risk of incident CHD, including sympathetic hyperactivity, systemic inflammation, oxidative stress, vascular endothelial dysfunction, and hypercoagulability [13, 14]. Likewise, sympathetic hyperactivity and systemic inflammation, but also elevated cortisol (COR) levels have been proposed to be biological mechanisms potentially linking insomnia with incident CVD [15]. Although these mechanisms might also link poor sleep with recurrent atherothrombotic events in patents with established CHD, this has not systematically been explored. In the present study, we focused on circulating neuroendocrine and coagulation markers in patients with sleep problems admitted with acute MI, as sleep studies in individuals without CVD suggest a role of neuroendocrine changes and coagulation activation in incident atherothrombotic events. For instance, in middle-aged and elderly subjects, objective sleep problems, including greater apnea-hypopnea index and oxygen desaturation, indicating OSA, as well as poor subjective sleep quality, were associated with increased plasma levels of fibrinogen, D-dimer, and von Willebrand factor (VWF) [16–20]. In patients with ACS, these prothrombotic markers were of prognostic value for major cardiovascular events and all-cause mortality [21–24].

In OSA, hyperactivity of the sympathetic nervous system (SNS) is evidenced by increased norepinephrine (NEPI) turnover [25], whereas findings on hypothalamic-pituitary adrenal (HPA) axis function, including COR levels, are contradictory [26]. In insomnia, HPA axis dysregulation is evidenced by high COR levels in the evening, affecting sleep architecture [27], and low morning COR levels, which relate to poor sleep quality, nightly awakenings and unrefreshing sleep [28]. The metabolic consequences of neuroendocrine dysregulation include hypertension, dyslipidemia, obesity, diabetes and a prothrombotic state, which are associated with the progression of atherothrombotic disease [29]. Secretion of epinephrine (EPI), NEPI, and COR has effects on coagulation, including increases in fibrinogen, D-dimer and VWF levels [30, 31], and neuroendocrine dysregulation might partially account for a link between sleep problems and coagulation activity [17].

Insomnia and OSA are often found together in the same patient, with up to 50% of OSA patients having insomnia symptoms; a similar prevalence of at least mild OSA is found in patients with insomnia [32]. However, in meta-analyses of prospective studies incident and recurrent CVD risk were not adjusted for the other sleep disorder nor were joint effects investigated [3–9]. As yet, except for hypertension [33], there is little evidence that OSA and insomnia together are associated with a greater risk of CVD or frequency of CVD risk factors than OSA alone [34]. However, such studies are still few in number and, to our knowledge, researchers did not investigate neuroendocrine and coagulation activity.

In this study we aimed to examine the association of a high risk of OSA and insomnia symptoms, independently of each other, with neuroendocrine hormones and coagulation factors in patients with acute MI. We specifically hypothesized that more sleep problems would be associated with higher levels of fibrinogen, D-dimer and VWF. We specified no a priori hypothesis regarding the direction of an association of sleep problems with EPI, NEPI and COR levels; this is because findings vary across studies, depending upon circadian factors, natural vs. experimental designs, confounding variables, and measures of sleep quantity and quality [26, 35, 36]. We predicted the hypothesized relationships to be independent of demographics, comorbidity, health behaviors, and cardiac-related variables. In complementary analyses we examined whether neuroendocrine hormones may account for the relation between sleep problems and coagulation factors, and whether there were joint effects of OSA risk and insomnia symptoms for neuroendocrine and coagulation outcomes.

## Methods

### Study participants and design

#### Rationale of the parent study

Data for this ancillary sleep study were collected from patients who participated in the Myocardial Infarction-Stress Prevention Intervention (MI-SPRINT) randomized controlled trial [37]. The primary aim of the parent study was to test whether a psychological first aid approach (i.e., one single 45-min session of psychological counseling at hospital referral) may prevent the incidence of ACS-induced posttraumatic stress at 3-month follow-up [37]. About 12% of ACS patients develop clinically significant levels of posttraumatic stress which is a predictor of poor cardiac prognosis [38]. Disrupted sleep could be one mechanism to explain this link [39]. The overarching hypothesis was that patients undergoing trauma-focused counseling would show better mental and physical health, including sleep, at follow-up than patients undergoing general stress counseling [37]. The intervention was not considered for the present study, as it showed no significant association with sleep, neuroendocrine and coagulation outcomes at hospital admission.

#### Patient recruitment

In brief, for MI-SPRINT, we recruited 190 patients with verified acute ST-elevation MI (STEMI) or non-STEMI and referred for acute coronary care intervention to the Bern University Hospital (“Inselspital”) between January 2013 and September 2015. The diagnostic criteria and therapy for acute STEMI and non-STEMI followed the European Society of Cardiology guidelines on myocardial revascularization, including indications for primary percutaneous coronary intervention in non-STEMI, antithrombotic treatments, and treatments to establish stable hemodynamic conditions [40, 41]. Inclusion criteria were 18 years or older, stable hemodynamic conditions, and a high level of acute distress during MI defined by scores of at least 5 for chest pain plus at least 5 for fear of dying and/or helplessness on numeric rating scales from 0 to 10 [37]. Exclusion criteria were emergency coronary artery bypass grafting, comorbid diseases likely to cause death within 1 year, cognitive impairment, severe clinical depression, suicidal ideations in the prior 2 weeks, inadequate knowledge of German, or participation in another trial. The study protocol was approved by the ethics committee of the State of Bern (KEK-Nr. 170/12). All patients were informed and gave signed consent.

#### Data collection

Within 48 h after having reached stable hemodynamic conditions, all included patients underwent a structured clinical interview on the coronary care unit on which occasion a medical history, sleep-related data and information on health behaviors were collected. To limit patient burden in this acute clinical setting, we decided to assess sleep problems with just two short screening tools, one to rate OSA risk, and one to rate the severity of sleep difficulties in terms of insomnia symptoms (see below). Cardiac-related variables were additionally abstracted from hospital charts. Fasting venous blood samples were collected for the measurement of neuroendocrine hormones and coagulation factors the next morning at 6 am. For logistical reasons and reasons of patient care, blood was collected non-fasting in 12 cases and not at 6 am in 46 cases. Specific collection times were between 1 am and 5 am (*n* = 14), at 6 am (*n* = 144), between 7 am and 8 am (*n* = 13), between 9 am and 1 pm (*n* = 10), and between 2 pm and 6 pm (*n* = 9). Collection time (all *p*-values > 0.64) and fasting state (all p-values > 0.05) were not significantly associated with circulating levels of any neuroendocrine hormone or coagulation factor.

### Measures

#### Sleep problems

To rate the risk of OSA, we applied a slightly modified version of the STOP questionnaire that is a tool to screen patients for OSA, asking for snoring (S); tiredness (T), observed (O) breathing cessations during sleep; and high blood pressure (P) [42]. The STOP questionnaire has been validated in patients with CHD [43] and in German [44]. The STOP questionnaire is particularly sensitive to identify patients with more severe forms of OSA [45], including in CHD patients [43]. Specific questions in our study (slight modifications from the original tool) asked about a history of snoring (irrespective of its loudness); history of breathing cessations while sleeping (although not necessarily observed); being often fatigued after sleep or while awake (sparing sleepiness); and history of hypertension (sparing treatment). Patients answering “yes” to at least two of these four questions were classified with a “high OSA risk”, and all others with a “low OSA risk” [42]. To verify the accuracy of the STOP screening tool to a certain extent, we also inquired about a positive history of sleep apnea, predicting that patients who confirmed would largely fall into the group with a high OSA risk. We also asked patients whether they lived with someone to account for the fact that household members often notice and communicate signs of sleep-disordered breathing. Patients identified with a high OSA risk were not treated for a presumed sleep disorder during their acute hospitalization.

Sleep difficulties in terms of insomnia symptoms in the previous 4 weeks were inquired with the 4-item Jenkins Sleep Scale with questions about difficulties initiating sleep, frequent awakenings during the night, difficulties maintaining sleep, and non-restorative sleep (i.e., feeling tired and worn out in the morning after a usual night’s sleep) [46]. Response alternatives (scores) are: not at all (0), 1–3 days (1), 4–7 days (2), 8–14 days (3), 15–21 days (4), and 22–31 days (5), yielding an average total score of all sleep difficulties between 0 and 5. Lower scores are indicative of better sleep quality. For illustrative purposes, patients were categorized in those with poor (sore > 2) vs. good (score ≤ 2) sleep. Cronbach’s alpha was 0.70 in the present study, suggesting acceptable internal consistency of the scale.

#### Neuroendocrine hormones

Serum COR (nmol/L) was determined with an electro-chemiluminescence immunoassay on a Cobas analyzer (Roche Diagnostics, Switzerland) at the Institute of Clinical Chemistry, Inselspital, Bern University Hospital, Switzerland. EPI and NEPI concentrations were quantified in EDTA plasma by high-pressure liquid chromatography using electrochemical detection [47] at the Laboratory for Stress Monitoring, Göttingen, Germany (inter−/intra-assay coefficients of variation < 10%; limit of detection 10 pg/mL). Undetectable EPI levels (*n* = 16) were assigned half the limit of detection (5 pg/mL).

#### Coagulation factors

Following a strict in-house protocol to ensure adequate preanalytical conditions, all coagulation factors were determined in citrate plasma at the haemostasis laboratory, Inselspital, Bern University Hospital, Switzerland. Fibrinogen measurements (g/L) were performed according to the Clauss method (Dade® Thrombin, Dade Behring, Liederbach, Germany). Plasma D-dimer levels (ng/mL) were determined with a quantitative sandwich enzyme immunoassay (VIDAS-D-Dimer, bioMérieux, Geneva, Switzerland), and from June 2015 on, with a particle-based immunoturbidimetric assay (Innovance® D-Dimer; Siemens AG, Munich, Germany). A highly significant correlation (Passing Bablock regression analysis: *r* = 0.952) between D-dimer values assessed with these two methods has previously been demonstrated, suggesting that their analytical performance is equally good [48]. Von Willebrand factor antigen concentration (IU/dL) was determined with vWF Ag® Kit (Siemens AG, Munich, Germany), an immunoturbidimetric method using polystyrene-based antibodies against VWF.

#### Covariates

In order to avoid overfitting of multivariate models, we included a selection of 16 potentially relevant predictors that might be confounders of the relationship between sleep problems and neuroendocrine measures and coagulation factors. We selected these covariates a priori based on the literature and on theoretical assumptions.

#### Demographic factors

Age and gender were abstracted from hospital charts. Socioeconomic status was based on the highest education level (high: high school graduation/matura, university graduation, including applied sciences; medium: apprenticeship or vocational school; low: lower than apprenticeship or vocational school) [49].

#### Comorbidities

We calculated the Charlson comorbidity index that provides an estimate of future mortality across several diseases [50]. Information on high cholesterol, hypertension, diabetes either with or without end organ damage, and lifetime depression was obtained through history taking.

#### Health behaviors

Patients disclosed their weight and height for the calculation of the body mass index. Smoking status was assessed in terms of current, former and never smokers, and physical activity (“that makes you sweat^”^) in terms of the average frequency per week. Regarding alcohol consumption, we categorized patients into abstainers, moderate drinkers, and heavy drinkers (> 21 drinks/week for men, > 14 drinks/week for women).

#### Cardiac-related variables

These included the type of MI (STEMI/non-STEMI), previous MI (yes/no), and the number of coronary arteries with stenosis ≥50%. As an objective marker of MI severity, we calculated the Global Registry of Acute Coronary Events (GRACE) risk score from eight variables obtained at hospital admission: age, heart rate, systolic blood pressure, creatinine, Killip class, cardiac arrest, ST-segment deviation, and elevated cardiac enzymes [51]. The GRACE score is a robust predictor of the cumulative risk of death or death and recurrent MI from admission to 6 months after discharge [51].

#### Acute distress

The intensity of perceived distress during MI was assessed with three numeric rating scales (scores 0–10) for “pain intensity (during MI)”, “fear of dying (until admission to the coronary care unit)” and “making sorrows and feeling helpless (when being told about having MI)” [37]. These measures were previously shown to predict CVD-related hospital readmissions in ACS patients after a 3-year follow-up [52]. The added sum score of the three numeric rating scales was divided by three to yield a severity index of acute distress.

### Data analysis

Data were analyzed using SPSS 23.0 for Windows (SPSS Inc., Chicago, IL) with level of significance at *p* < 0.05. For technical and logistic reasons (e.g., early discharge of patients), EPI and NEPI were missing in 45 patients, COR in 16 patients, fibrinogen in 14 patients, and D-dimer and VWF in 15 patients each. Due to lacking information, the GRACE risk score could not be computed for 18 patients. Seven or less values were missing for all other measures. We replaced all missing values with the expectation maximization algorithm to make use of all the available information from the total sample of 190 study participants. Supporting the adequacy of this approach, the strength and significance of the associations between OSA risk and COR (*r* = − 0.16, *p* = 0.043; *n* = 170) and between sleep difficulties and both fibrinogen (*r* = 0.16, *p* = 0.032; *n* = 172) and NEPI (*r* = − 0.18, *p* = 0.036; *n* = 141) in the patients with complete data for these measures were similar to those shown in the result section for the whole sample. Values of neuroendocrine hormones and coagulation factors were log-transformed before analysis.

Pearson correlation analysis was used to estimate the univariate relationship between two variables. Multivariate regression analysis was employed to identify whether OSA risk (categorical variable: high vs. low) and sleep difficulties (continuously scaled variable) were independently associated with neuroendocrine hormones and coagulation factors, after controlling for demographics, comorbidity, health behaviors, and cardiac-related variables, all entered in one block. If the total sleep difficultly scale showed a significant association, we computed post hoc analyses on each individual item. Finally, to test for a possible joint effect of OSA risk and sleep difficulties on outcomes, we additionally entered the interaction between the STOP score and the sleep difficulties score total into the regression equation. We allowed a maximum of 19 independent predicting variables to protect against model overfitting. Cook’s distance and variance inflation factor, respectively, were used to verify the absence of influential outliers in the set of predictor variables and critical multicollinearity between two predictor variables, respectively.

We did not adjust *p*-values for multiple comparisons because of the pre-established primary hypothesis of a significant association of sleep problems with neuroendocrine and coagulation measures, and because of the risk of deeming truly important differences non-significant in a still nascent field of research [53]. However, partial correlation coefficients (r_p_) from the regression output were used to interpret effect sizes as small (0.1), medium (0.3), or large (0.5), irrespective of p-values [54].

## Results

### Patient characteristics

Table 1 shows the characteristics of the 190 patients, all of Caucasian ethnicity. The majority of participants were well-educated men with a first-time STEMI and living together with someone. The Charlson index indicated low comorbidity; nevertheless, a history of hypertension and high cholesterol were reported by every other patient, and a history of depression by almost 30%. According to the GRACE score, the median (inter-quartile range) risk of 6-month death was 5% (3–9). Regarding health behaviors, patients were on average overweight, and a substantial portion were current smokers and physically inactive. Defining the group of poor sleepers, a total of 52 (27.4%) patients reported sleep difficulties on more than 7 days in the previous 4 weeks. A total of 77 (40.5%) patients could be identified with a high OSA risk; 15 (78.9%) of the 19 patients with a history of sleep apnea were in this group. Living with someone was not associated with OSA risk (*p* = 0.98), so it was not used as a covariate. The type of drugs delivered at admission and glucocorticoid use did not significantly differ between patients with high versus low OSA risk (all *p*-values > 0.06).

**Table 1:** Demographic and clinical characteristics of the 190 study participants

Age, yrs., M (SD)	59.9 (11.2)
Sex (men), *n* (%)	157 (82.6)
Education level	
High, *n* (%)	36 (18.9)
Medium, n (%)	136 (71.6)
Low, *n* (%)	18 (9.5)
Living with someone, *n* (%)	138 (72.6)
Charlson comorbidity index, M (SD)	1.80 (1.20)
Positive history of hypertension, *n* (%)	98 (51.6)
Positive history of high cholesterol, *n* (%)	86 (45.3)
Positive history of depression, *n* (%)	54 (28.4)
Body mass index, kg/m^2^, M (SD)	27.7 (4.6)
Smoking status	
Current smoker, *n* (%)	83 (43.7)
Former smoker, *n* (%)	50 (26.3)
Never smoker, *n* (%)	57 (30.0)
Alcohol consumption (drinks per week)	
Abstainers, *n* (%)	33 (17.4)
Moderate drinkers, *n* (%)	147 (77.4)
Heavy drinkers, *n* (%)	10 (5.3)
Physical activity (number of times per week)	
3–7, *n* (%)	49 (25.8)
1–2, *n* (%)	52 (27.4)
< 1, *n* (%)	89 (46.8)
Previous myocardial infarction, *n* (%)	20 (10.5)
Previous percutaneous coronary intervention	27 (14.2)
Previous coronary artery bypass surgery	5 (2.6)
Type of confirmed acute infarction	
ST-elevation myocardial infarction, *n* (%)	136 (71.6)
Non-ST-elevation myocardial infarction, *n* (%)	54 (28.4)
Major coronary arteries with stenosis ≥50% (number), M (SD)	1.93 (0.86)
Left ventricular ejection fraction (angiography), %, M (SD)	47. 6 (11.8)
Vasopressant drugs at admission	
Epinephrine, *n* (%)	9 (4.7)
Norepinephrine, *n* (%)	6 (3.2)
Dobutamine, *n* (%)	8 (4.2)
Dopamine, *n* (%)	1 (0.5)
Antithrombotic therapy at admission	
Aspirin, *n* (%)	190 (100)
Clopidogrel, *n* (%)	57 (30.0)
Prasugrel, *n* (%)	99 (52.1)
Ticagrelor, *n* (%)	80 (42.1)
Fondaparinux, *n* (%)	154 (81.1)
Unfractioned heparin, *n* (%)	145 (76.3)
Low molecular weight heparin, *n* (%)	13 (6.8)
Bivalirudin, *n* (%)	2 (1.1)
Abciximab, *n* (%)	46 (24.2)
Thrombolysis, *n* (%)	2 (1.1)
Beta-blocker therapy at admission, *n* (%)	173 (91.1)
Regular use of glucocorticoids, *n* (%)	13 (6.8)
Global Registry of Acute Coronary Events risk score, M (SD)	107 (27)
Acute distress during myocardial infarction, M (SD)	6.23 (1.40)
Sleep difficulties total, M (SD)	1.53 (1.29)
Difficulties initiating sleep, M (SD)	1.24 (1.62)
Nighttime awakenings, M (SD)	2.53 (2.10)
Difficulties maintaining sleep, M (SD)	1.17 (1.68)
Nonrestorative sleep, M (SD)	1.17 (1.67)
High risk of obstructive sleep apnea, *n* (%)	77 (40.5)

### Unadjusted associations among sleep, neuroendocrine and coagulation measures

There were several significant bivariate relationships between sleep problems, neuroendocrine and coagulation measures. High OSA risk was associated with more sleep difficulties (*r* = 0.20, *p* = 0.006), with frequent nighttime awakenings (*r* = 0.30, *p* < 0.001) and nonrestorative sleep (*r* = 0.19, *p* = 0.011) driving this relationship. High OSA risk was also associated with lower COR levels (*r* = − 0.17, *p* = 0.022), and more sleep difficulties were associated with higher fibrinogen levels (*r* = 0.14, *p* = 0.048), with difficulties initiating sleep driving this relationship (*r* = 0.22, *p* = 0.003).

Further expected direct associations were seen among all stress hormones (EPI and NEPI: *r* = 0.51; EPI and COR: r = 0.30; NEPI and COR: 0.29; *p*-values < 0.001) and among all coagulation factors (fibrinogen and D-dimer: *r* = 0.35; fibrinogen and VWF; *r* = 0.38; D-dimer and VWF: r = 0.38; p-values < 0.001). Direct associations also emerged for NEPI with D-dimer (*r* = 0.17, *p* = 0.020) and VWF (*r* = 0.16, *p* = 0.033) levels, and between COR and fibrinogen levels (*r* = 0.21, *p* = 0.004).

### Independent associations of sleep measures with neuroendocrine hormones

**Fig. 1:**
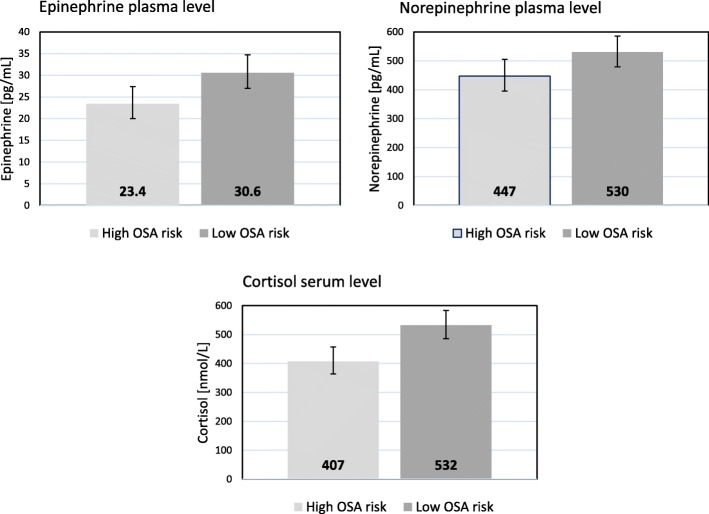
Risk of obstructive sleep apnea and mean values of neuroendocrine measures. The bar graphs illustrate the significant differences in epinephrine (*p* = 0.015), norepinephrine (*p* = 0.049) and cortisol (*p* = 0.001) levels between subjects with high (*n* = 77) vs. low (*n* = 113) risk of obstructive sleep apnea (OSA), expressed as geometric mean values with 95% confidence interval. Adjustments were made for age, sex, education, Charlson comorbidity index, positive history of hypertension/high cholesterol/depression, body mass index, smoking, alcohol consumption, physical activity, previous myocardial infarction (MI), ST-segment MI, number of stenotic coronary arteries, Global Registry of Acute Coronary Events risk score, acute distress, and sleep difficulties

**Fig. 2:**
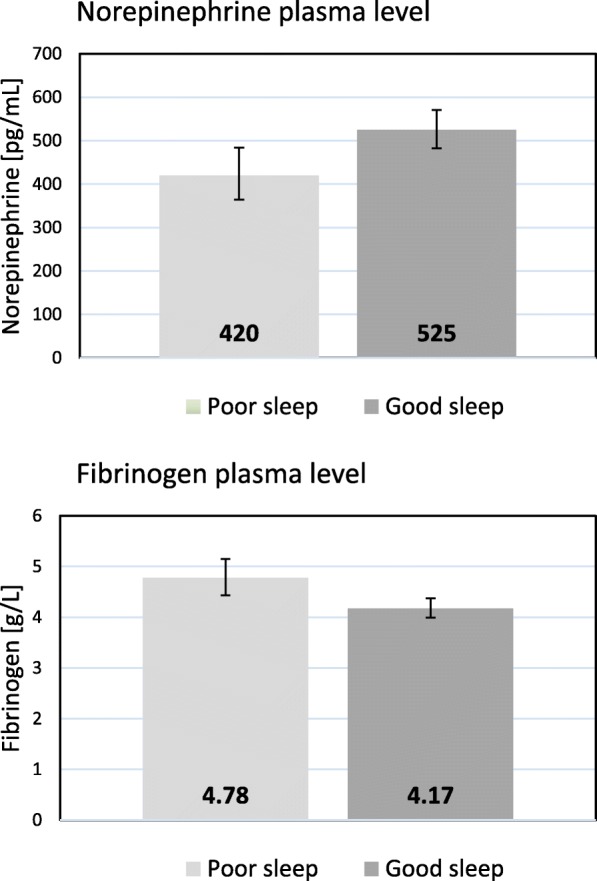
Sleep difficulties and mean values of neuroendocrine and coagulation measures. The bar graphs illustrate the significant differences in norepinephrine (*p* = 0.010) and fibrinogen (*p* = 0.003) levels between subjects with poor sleep (i.e., sleep difficulties on more than 7 days in the previous 4 weeks; *n* = 52) and those with good sleep (*n* = 138), expressed as geometric mean values with 95% confidence interval. Adjustments were made for age, sex, education, Charlson comorbidity index, positive history of hypertension/high cholesterol/depression, body mass index, smoking, alcohol consumption, physical activity, previous myocardial infarction (MI), ST-segment MI, number of stenotic coronary arteries, Global Registry of Acute Coronary Events risk score, acute distress, and risk of obstructive sleep apnea

Table 2 shows the fully adjusted multivariate model for the levels of neuroendocrine hormones. Compared to patients with low OSA risk, those with high OSA risk had significantly lower levels for all three hormones. Figure 1 shows absolute values of these group differences. More sleep difficulties (continuously scaled) were also significantly associated with lower NEPI levels (absolute values illustrated in Fig. 2 for poor vs. good sleepers). The relationship between sleep difficulties and NEPI was driven by difficulties initiating (r_p_ = − 0.15, *p* = 0.049) and maintaining (r_p_ = − 0.20, *p* = 0.008) sleep. Moreover, there was no significant interaction between OSA risk and sleep difficulties for stress hormones (*p*-values > 0.36).

**Table 2:** Multivariate relations between sleep problems and neuroendocrine measures

Entered variables	Epinephrine		Norepinephrine		Cortisol	
	Partial corr.	*P*	Partial corr.	*P*	Partial corr.	*P*
Age	**−0.183**	**0.016**	0.050	0.516	−0.087	0.254
Male sex	−0.079	0.302	0.080	0.295	−0.031	0.690
Education	−0.092	0.230	−0.003	0.970	−0.051	0.504
Charlson index	**−0.240**	**0.001**	−0.126	0.098	−0.111	0.147
Hypertension history	**0.159**	**0.037**	0.088	0.247	0.139	0.068
High cholesterol history	0.130	0.089	−0.070	0.357	− 0.002	0.978
Depression history	0.070	0.360	0.054	0.479	0.007	0.928
Body mass index	−0.053	0.489	**0.178**	**0.019**	0.101	0.187
Smoking	−0.047	0.541	−0.021	0.786	0.030	0.693
Alcohol consumption	0.026	0.731	−0.126	0.098	0.025	0.747
Physical activity	−0.149	0.051	−0.031	0.688	0.043	0.572
Previous MI	0.140	0.067	0.001	0.994	0.074	0.333
ST-elevation MI	**0.150**	**0.049**	**0.172**	**0.024**	0.081	0.289
Stenotic coronary arteries	0.074	0.334	0.116	0.130	**0.232**	**0.002**
GRACE risk score	**0.234**	**0.002**	0.131	0.085	**0.157**	**0.039**
Acute distress	**0.210**	**0.006**	**0.170**	**0.025**	0.029	0.706
Sleep difficulties	−0.054	0.482	**−0.172**	**0.024**	0.094	0.217
High risk of OSA	**−0.185**	**0.015**	**−0.150**	**0.049**	**−0.251**	**0.001**
Model statistic	R^2^ = 0.295 F_18,171_ = 3.97 *P* < 0.001		R^2^ = 0.212 F_18,171_ = 2.55 *P* = 0.001		R^2^ = 0.174 F_18,171_ = 2.00 *P* = 0.012	

Linear regression model with all variables entered in one block. Significant correlation coefficients (Partial corr.) and *P*-values are given in bold

*GRACE* Global Registry of Acute Cardiac Events, *MI* myocardial infarction, *OSA* obstructive sleep apnea

### Independent associations of sleep measures with coagulation factors

Table 3 shows the fully adjusted multivariate model for the three coagulation factors. More sleep difficulties were associated with greater fibrinogen levels (absolute values illustrated in Fig. 2 for poor vs. good sleepers), with difficulties initiating sleep driving this relationship (r_p_ = 0.25, *p* = 0.001). Risk of OSA was not significantly associated with any coagulation factor. Likewise, there was no significant interaction between OSA risk and sleep difficulties for coagulation factors (*p*-values > 0.35).

**Table 3:** Multivariate relationship between sleep problems and coagulation factors

Entered variables	Fibrinogen		D-dimer		VWF	
	Partial corr.	*P*	Partial corr.	*P*	Partial corr.	*P*
Age	−0.059	0.439	−0.125	0.102	−0.006	0.942
Male sex	−0.073	0.343	−0.083	0.278	−0.114	0.137
Education	**−0.190**	**0.012**	−0.096	0.208	−0.018	0.810
Charlson index	0.115	0.133	**0.177**	**0.020**	0.021	0.779
Hypertension history	0.078	0.309	0.037	0.628	−0.034	0.654
High cholesterol history	0.041	0.591	−0.063	0.409	−0.049	0.522
Depression history	**−0.178**	**0.019**	0.037	0.627	−0.060	0.432
Body mass index	0.133	0.081	−0.011	0.890	0.136	0.075
Smoking	0.032	0.678	−0.061	0.426	0.003	0.973
Alcohol consumption	−0.054	0.477	0.087	0.253	0.032	0.674
Physical activity	−0.090	0.238	0.069	0.367	−0.012	0.877
Previous MI	0.047	0.538	**0.155**	**0.042**	0.082	0.286
ST-elevation MI	**0.158**	**0.038**	0.019	0.800	0.010	0.898
Stenotic coronary arteries	0.074	0.331	−0.083	0.280	0.003	0.972
GRACE risk score	0.102	0.180	**0.279**	**< 0.001**	**0.276**	**< 0.001**
Acute distress	0.033	0.668	0.060	0.430	0.126	0.097
Sleep difficulties	**0.158**	**0.037**	0.040	0.600	0.079	0.299
High risk of OSA	−0.038	0.618	−0.082	0.283	0.017	0.828
Model statistic	R^2^ = 0.216 F_18,171_ = 2.61 *P* = 0.001		R^2^ = 0.245 F_18,171_ = 3.08 *P* < 0.001		R^2^ = 0.236 F_18,171_ = 2.93 *P* < 0.001	

Linear regression model with all variables entered in one block. Significant partial correlation coefficients (Partial corr.) and *P*-values are given in bold

*GRACE* Global Registry of Acute Cardiac Events, *MI* myocardial infarction, *OSA* obstructive sleep apnea, *VWF* von Willebrand factor

### Role of stress hormones in the relation between sleep and coagulation factors

The positive association of sleep difficulties with fibrinogen levels was virtually unchanged when EPI (r_p_ = 0.16, *p* = 0.043), NEPI (r_p_ = 0.16, *p* = 0.031) and COR (r_p_ = 0.15, *p* = 0.055) were separately added to the model in Table 3. Neuroendocrine hormones were not significantly predictive of fibrinogen (*p*-values > 0.06), D-dimer (*p*-values > 0.07) and VWF (*p*-values > 0.37) levels, independently of all other covariates. Also, OSA risk (*p*-values > 0.27) and sleep difficulties (*p*-values > 0.11) showed no significant interaction with neuroendocrine hormones for any coagulation factor.

## Discussion

In patients admitted with acute MI, we found a high risk of OSA to be associated with decreased levels of EPI, NEPI, and COR, compared to when OSA risk was low, independent of covariates. Sleep difficulties in terms of insomnia symptoms were also independently associated with low NEPI levels, although not with EPI and COR levels. Less evidence was found for an independent relationship between sleep problems and coagulation factors. Whereas insomnia symptoms were associated with higher levels of fibrinogen, although not with D-dimer and VWF, OSA risk did not show a significant association with any coagulation factor. The size of these associations was similar to that observed for several covariates with neuroendocrine and coagulation outcomes, including the prognostic GRACE score, implying not only statistical, but also clinical significance with small-to-moderate effects of sleep problems.

In our sample, 40.5% of patients had a high OSA risk, a prevalence similar to previous studies showing that close to a half of patients with ACS have moderate or severe OSA (apnea-hypopnea index ≥15) [55]. Also comparable to a previous study showing that 37.3% of hospitalized patients with ACS have clinically relevant insomnia symptoms [56], 27.4% of our patients were poor sleepers. In agreement with previous studies on CVD outcomes [34], we did not observe joint effects between OSA risk and insomnia symptoms for neuroendocrine hormones and coagulation factors. However, concurring with a high prevalence of OSA among insomnia patients and vice versa [32], we found a significant association between OSA risk and insomnia symptoms with frequent awakenings and non-restorative sleep driving this association. As we adjusted for an overlap between OSA risk and insomnia symptoms, we may interpret that in the setting of acute MI, neuroendocrine and coagulation abnormalities differ substantially between high OSA risk and insomnia symptoms and even between individual insomnia symptoms. We particularly found difficulties in initiating sleep to be significantly associated with NEPI and fibrinogen, and difficulties maintaining sleep also with NEPI, whereas waking up during the night and nonrestorative sleep showed no association with neuroendocrine hormones and coagulation factors whatsoever.

Increased fibrinogen levels in acute MI patients with more severe insomnia symptoms is compatible with a prothrombotic state, one mechanism that may possibly increase the risk of recurrent atherothrombotic events [21]. In addition to having rheological and hemostatic properties, fibrinogen is also an inflammatory molecule (i.e., an acute phase reactant), and inflammation and sleep disturbances are reciprocally linked with each other [57]. However, as we measured fibrinogen only once, but inquired about insomnia symptoms covering the preceding 4 weeks, we are unable to make any causal inferences. For instance, it is possible that insomnia symptoms were already associated with elevated fibrinogen before MI and/or even a contributing factor to MI onset, although exaggerated fibrinogen production during the acute phase could also have occurred in those with more severe insomnia symptoms. Clearly, our finding in fibrinogen needs replication, as we did not observe a relation between insomnia symptoms and the other two prothrombotic markers. Future studies should also include measures of impaired fibrinolysis, particularly plasminogen activator inhibitor-1 [16, 19].

Regarding neuroendocrine dysregulation, low endogenous COR levels in CHD patients with high OSA risk could result in less curtailing of vascular inflammation and atherosclerosis progression, respectively [58]. Indeed, low cortisol levels at admission were previously shown to predict early death in patients with acute MI [59]. Likewise, low catecholamine levels, found in those with high OSA risk and more sleep difficulties in our study, are implied in increased systemic inflammation [60]. Moreover, in OSA, due to the accompanying SNS hyperactivity, beta-2-adrenergic receptors are desensitized [61], a mechanism that may result in blunted beta-2-adrenergic receptor-mediated anti-inflammatory effects of catecholamines [60]. As coagulation activation relates to catecholamine and cortisol activity [30, 31], comparably low SNS and HPA activation in relation to sleep problems may help to explain why neuroendocrine dysregulation was no associated with coagulation factors in patients with high OSA risk and insomnia symptoms, respectively.

That patients with OSA might not mount an appropriate stress response to ACS could not only have deleterious consequences for cardiac prognosis, as there is data from animal models to suggest that chronic intermittent hypoxia is a potentially adaptive stimulus for the myocardium in OSA [62]. One study in humans found more severe OSA to be associated with less cardiac injury during non-fatal ACS, proposing that ischemic preconditioning due to chronic intermittent hypoxia could be a possible explanation [63]. Although the involved physiological mechanisms are still elusive, there may be pro-angiogenic and anti-oxidant effects of intermittent hypoxia [62, 63]. For instance, as increased sympathetic tone and elevated catecholamine levels in OSA might be associated with increased reactive oxygen species production [64]. A lower sympathoadrenal response in the setting of ACS might result in less oxidative stress and myocardial damage, respectively.

Our study has several limitations worth mentioning. Most importantly, the lack of objective measures of sleep architecture, sleep duration and sleep apnea testing does not allow us to draw firm conclusions. To better differentiate between effects of OSA risk and insomnia symptoms, polysomnography or the additional inclusion of validated screening tools, such as the Berlin questionnaire and the Epworth Sleepiness Scale would have yielded data that are more reliable. We were particularly unable to tease apart influences of obstructive versus central sleep apnea that is prevalent in ACS. Moreover, objective short sleep duration is an important factor driving activation of the stress system and cardiometabolic morbidity in patients with insomnia [65]. However, it should also be mentioned that the acute cardiac situation influences sleep macro- and microarchitecture [66], which can complicate the diagnostic process. The STOP screening questionnaire is not very sensitive and specific for moderate-to-severe sleep-disordered breathing in CHD [42], but also in other disorders [42, 67] and in population-based studies [45]. A high sensitivity to detect more severe forms of OSA in patients with coronary artery bypass surgery was associated with low specificity [42], which increases the risk of false positives. The STOP questionnaire does not allow a firm assessment of OSA severity. Follow-up sleep studies would have been necessary for this purpose. It is not clear how the modified STOP questionnaire influenced its sensitivity and specificity. The omission of loudness of snoring may have overestimated the prevalence of patients classified with high OSA risk. Nonetheless, the majority of patients who reported a history of sleep apnea were in this group, suggesting our slightly modified STOP tool showed clinically useful sensitivity. Although time of blood collection did not significantly relate to coagulation and neuroendocrine measures, given their circadian variation, the difference in the time of assessment is a limitation. Although we applied an established method to replace missing data, the fact that 24% of patients missed catecholamine measures is a further limitation. We controlled for a range of important covariates, but residual confounding through for instance combined effects of emergency medications remains a possibility. This weakens the interpretation of the relationships which only emerged as significant in the covariate-adjusted analyses. In turn, non-significant results must be interpreted with caution, as we did not perform a power calculation for this ancillary sleep study. Body mass index, a driver of neuroendocrine and coagulation measures, was self-reported. Although we controlled for acute distress during MI, the specifics of our sample with substantially distressed patients need to be considered when generalizing the findings to other cohorts of ACS patients. Distressed patients may have over-reported sleep difficulties in their effort to make sense of the situation. However, about 70% of patients experience at least moderate distress and fear of dying during ACS [68]. As is common in ACS studies, less than 20% of our patients were women, which precluded sex-stratified analyses, which might result in different findings for women and men [10, 11].

## Conclusions

In patients admitted with acute MI, high OSA risk and insomnia symptoms might differently affect neuroendocrine and coagulation activity. While a high OSA risk was associated with neuroendocrine dysregulation, indicating SNS and HPA axis hypoactivity, insomnia symptoms, particularly problems initiating or maintaining sleep, showed a relation with reduced SNS activity and a prothrombotic state. Low cortisol and catecholamine levels might result in less curtailing of vascular inflammation, and elevated fibrinogen levels might evoke a prothrombotic state, with such pathophysiology helping to explain adverse outcome of MI patients with sleep problems. In turn, the finding of lower neuroendocrine hormones in patients with acute MI and a high OSA risk may also inform further studies to better understand potentially cardioprotective mechanisms of chronic intermittent hypoxia.

## Abbreviations

ACS: Acute coronary syndrome
CHD: Coronary heart disease
COR: Cortisol
CVD: Cardiovascular disease
EPI: Epinephrine
GRACE: Global Registry of Acute Cardiac Events
HPA: Hypothalamic-pituitary adrenal
MI: Myocardial infarction
NEPI: Norepinephrine
OSA: Obstructive sleep apnea
SNS: Sympathetic nervous system
STEMI: ST-elevation myocardial infarction
VWF: Von Willebrand factor
